# Construction and Validation of Chicken Immune scFv Antibody Library against *Helicobacter pylori*

**DOI:** 10.3390/microorganisms12061148

**Published:** 2024-06-05

**Authors:** Yanan Gong, Xiaoli Chen, Jiaming Fan, Lu Sun, Lihua He, Hairui Wang, Xiaomei Yan, Jianzhong Zhang

**Affiliations:** National Key Laboratory of Intelligent Tracking and Forecasting for Infectious Diseases, National Institute for Communicable Disease Control and Prevention, Chinese Center for Disease Control and Prevention, Beijing 102206, China; gongyanan@icdc.cn (Y.G.);

**Keywords:** *Helicobacter pylori*, chicken scFv, phage display, diagnosis

## Abstract

Accurate diagnostic techniques and effective therapeutic methods are required to treat *H. pylori*. The application of chicken single-chain variable fragment (scFv) antibodies may diagnose and treat *H. pylori*. This study used the phage display technique to construct a chicken-derived immune scFv antibody library against *H. pylori*. Total RNA was extracted from the spleens of five immunized chickens and reverse transcribed into cDNA. A fragment of scFv was produced by overlap extension PCR and cloned into a pHEN2 phagemid vector. After the package with the M13KO7 helper phage, the recombinant HpaA protein was used as a target antigen to validate the screening ability of our antibody library by bio-panning. The dilution counting results showed that the size of the primary antibody library was estimated to be 1 × 10^9^ cfu/mL. PCR analysis of 47 clones from the library revealed that about 100% of the clones were positive with scFv fragments, and there were no identical sequences, indicating the good diversity of the antibody library. After three rounds of bio-panning, high-affinity antibodies against recombinant HpaA protein were successfully obtained. The selected antibody specifically recognized HpaA protein in nine different *H. pylori* strains, confirming the screening ability of our library. The chicken immune scFv antibody library against *H. pylori* was successfully constructed, and the antibody library’s screening ability was validated by selecting specific scFv antibodies against recombinant HpaA and clinical strains. It provided a simple and rapid method to obtain antibodies against *H. pylori* for diagnosis or treatment.

## 1. Introduction

*Helicobacter pylori* (*H. pylori*) can colonize on the surface of gastric mucosa and cause serious gastrointestinal diseases, including chronic and atrophic gastritis, peptic ulcer, MALT lymphoma, and gastric cancer [1,2]. More than 90% of non-cardia stomach cancers worldwide are attributed to *H. pylori* infection [3]. Considering the disease burden caused by *H. pylori* infection, the current consensus recommends that eradication therapy should be taken once *H. pylori* infection is confirmed [4]. More accurate diagnostic techniques and effective therapeutic methods are required to treat *H. pylori*.

Antibodies, such as polyclonal or monoclonal antibodies, can be used in valuable therapeutic and diagnostic tools and have been introduced against *H. pylori* infection [5,6,7]. Several studies have been conducted to produce specific antibodies targeting different antigens of *H. pylori*, including the vacuolating toxin (VacA) and urease [8,9]. Chicken-derived antibodies, including egg yolk immunoglobulin Y (IgY), monoclonal, polyclonal, and scFv, have attracted increasing attention and have become ideal antibodies for *H. pylori* infection [10,11,12]. IgY has many advantages, such as a high yield, high binding specificity, and low cross-reactivity. It has been widely used to diagnose and treat gastrointestinal infections, including *H. pylori* [11,12,13,14]. scFv contains a complete antigen-binding site, and its size is one-sixth of an intact antibody, which makes gene manipulation easier. For scFv, the variable light chain (V_L_) and heavy chain (V_H_) of the antibody are connected by a short polypeptide linker to form a single-chain antibody [15]. Chicken scFv antibodies have been used in various infection diagnoses, such as *Staphylococcus aureus* [16,17,18]. For *H. pylori*, there was no study on screening for chicken-derived scFv antibodies.

Among various antibody development approaches, phage display is a powerful technique for producing antibodies against extensive antigens. Several phage display-based antibody fragments and epitopes have been identified against *H. pylori* [5,8,19]. Moreover, phage display has been widely used to select scFv antibodies with high specificity to the target antigens [20]. Therefore, we used the phage display technique in the present study to construct an immune chicken-derived scFv antibody library against *H. pylori*. We further verified the library by selecting specific anti-HpaA antibodies.

## 2. Materials and Methods

### 2.1. Bacteria Strains and Culture Conditions

The *H. pylori* strain 26695 and 9 clinical strains (numbered JX66, J20, ZSFY169, HB318, HB376, 14582, 30011, TJ945685, and 534) were stored in the National *H. pylori* Strain Bank of China in the National Institute for Communicable Disease Control and Prevention, Chinese Center for Disease Control and Prevention. All strains were separately streaked on Karmali agar plates supplemented with Karmali Agar base containing 15% defibrinated sheep blood, and the plates were incubated at 37 °C under microaerobic conditions (5%O_2_, 10% CO_2_, and 85% N_2_) for 2–3 days. Urease, oxidase, catalase tests, and bacterial morphology confirmed the obtained colony of each *H. pylori* strain.

The process of immunizing chickens (Jinghong No. 1 chickens) is described in our previous study [21]. This study was approved by the Ethics Committee of the National Institute for Communicable Disease Control and Prevention, Chinese Center for Disease Control and Prevention (2023-053).

### 2.2. Construction of the scFv Antibody Library

Total RNA was extracted from the spleens of five immunized chickens with TRIzol and reverse transcribed into cDNA using a Takara PrimeScript RT reagent kit (Takara, Japan) according to the manufacturer’s instructions. The V_H_ and V_L_ were amplified by PCR using the primers in Table 1. A 15-amino acid peptide linker connected the two domains to produce a full-length single-chain variable fragment (scFv) by overlap extension PCR with primer scFv-1. The amplification for scFv was performed as follows: 94 °C for 3 min, followed by 15 cycles of 94 °C for 15 s, 67 °C for 15 s, 72 °C for 45 s, and finally 72 °C for 3 min, which would yield a product size of 800 bp.

The scFv fragments were digested with restriction enzymes (*NcoI-NotI*) and cloned into a pHEN2 phagemid vector with the same restriction sites. The recombinant vectors were transformed into *E. coli* TG1 competent cells by electroporation (C = 25 μF, PC = 200 Ω, V = 2.5 kV). The size of the antibody library was calculated using the dilution counting method. The colonies on the plate were selected randomly, and PCR was performed using primer scFv-3. The PCR products were sequenced by the Sangon Company (Shanghai, China) to determine the diversity of the antibody library. The primary antibody library was stored at −80 °C.

A volume of 5 mL of *E. coli* cells of the primary library was inoculated into 1L 2 x YT broth (1.6% (*w*/*v*) tryptone, 1.0% (*w*/*v*) yeast extract, 0.5% (*w*/*v*) NaCl) supplemented with 100 μg/mL ampicillin and incubated until the OD_600_ reached 0.8. Approximately 10^12^ pfu M13KO7 (helper phage) was added into the culture and incubated overnight at 28 °C. After centrifugation (10,000 rpm, 10 min), the supernatant was precipitated with PEG/NaCl (20% PEG6000, 2.5 M NaCl) on ice for 4 h and centrifugated for 20 min (12,000 rpm). The precipitation was resuspended with PBS, and a volume of 20 μL phage suspension was taken to calculate the titer of the phage display library.

### 2.3. Expression and Purification of Recombinant HpaA Protein

The *hpaA* gene was amplified from the genome of *H. pylori* 26695 by PCR using primers in Table 1. The amplification was performed as follows: 94 °C for 5 min, followed by 35 cycles of 94 °C for 30 s, 55 °C for 30 s, and 72 °C for 45 s, and finally 72 °C for 5 min, which would yield a product size of 705 bp. The PCR product, as well as expression vector pET28a, were both digested with *BamHI* and *XhoI*. The ligation of digested products was performed at 25 °C for 1 h, and the recombinant plasmid was transformed into *E. coli* BL21(DE)_3_. The colonies growing on plates with kanamycin resistance were randomly selected for PCR. The expression of HpaA was induced by adding 0.5 mM isopropyl-β-D-thiogalactopyranoside (IPTG) and detected by SDS-PAGE.

The HpaA protein was purified with a HisTrap HP column (GE Healthcare, Chicago, IL, USA). Briefly, the bacterial cells were harvested via centrifugation at 8000 rpm for 30 min and resuspended with binding buffer (20 mM Tris-HCl, pH 8.0, 50 mM NaCl, 40 mM imidazole). After sonication, the protein was purified and eluted with elution buffer (20 mM Tris-HCl, pH 8.0, 50 mM NaCl, 500 mM imidazole). Protein concentration was determined using a BCA protein assay kit (Thermo Fisher, Waltham, MA, USA).

### 2.4. scFv Library Panning

HpaA-specific scFv was isolated from the phage display antibody library as follows: HpaA-coated plates (3 μg/well for the first round, 1.5 μg/well for the second round, and 750 ng/well for the third round) were blocked with PBS (2% skim milk powder), and approximately 10^12^ pfu phages were added into the plates. The plates were washed 9 times with PBST (0.1% (*v*/*v*) for the first round, 0.2% (*v*/*v*) for the second round, and 0.3% (*v*/*v*) for the third round) as well as once with PBS. The conjugated phage was eluted by adding 0.1 M Glycine (pH 2.2) and slowly shaken at room temperature for 20 min, then 1 M Tris buffer (pH 8.0) was added for quick neutralization. The eluted phages were amplified for the next round of panning. Briefly, log-phase TG1 cells were infected with eluted phages at 37 ℃ for 1 h, and then approximately 10^12^ pfu M13KO7 phages were added to co-culture for 1 h. After overnight culture at 30 °C, the supernatant was precipitated with 20% PEG/NaCl on ice for 4 h, and a volume of 20 μL phage suspension was taken to calculate the titer. Three rounds of bio-panning were performed to select scFv binding to HpaA.

### 2.5. Phage ELISA

The colonies were randomly selected, and their reactivity with HpaA was analyzed using phage ELISA. Briefly, a single colony was selected and incubated in the 96-well deep-well plate with 2 x YT medium until OD_600_ reached 0.8. The wells with no colony were used as the negative control. Approximately 10^12^ pfu M13KO7 phages were added into the deep-well plate and incubated at 30 ℃ for 16 h. The ELISA plates were coated overnight with HpaA protein (0.05 μg/well), and 100 μL culture supernatant from the deep-well plate was added to the ELISA plates for incubation. The bound phages were detected with 1:10,000 diluted HRP-conjugated mouse anti-M13 antibody (Abcam, Cambridge, MA, USA). The 3,3′,5,5′-tetramethylbenzidine (TMB) substrate solution was added to each well, and finally, the absorbance at 450 nm was measured. The HpaA-specific phage clones identified in phage ELISA were analyzed by DNA sequencing using primer scFv-3.

### 2.6. scFv Antibody Expression in P. pastoris and Purification

scFv antibody fragments were amplified from the positive colonies using primer scFv-2 and ligated into the multiple cloning sites of pICZαA (Invitrogen, Waltham, MA, USA) using *EcoRI* and *NotI* restriction sites. The ligation products were transformed into *E. coli* TOP10 strains and screened on LB plates containing 100 μg/mL zeocin (Thermo Fisher, Waltham, MA, USA).

The recombinant vectors isolated from positive colonies were linearized with *SacI* (NEB, Ipswich, MA, USA) and dephosphorylated with CIP (NEB, Ipswich, MA, USA). The final products were transformed into *P. pastoris* competent cell GS115 strains and selected on YPD plates containing 100 μg/mL zeocin. After incubation for 3 days at 30 °C, a single zeocin-resistant colony was selected for protein expression. Firstly, selected colonies were cultured in 5 mL BMGY medium containing 100 μg/mL zeocin overnight under shaking (200 rpm) at 30 °C. Then, 5 mL of yeast culture was transferred into 200 mL BMMY medium and cultured under shaking for 48 h, and 0.5% methanol was added to the medium every 24 h.

The supernatant of the culture was collected by centrifugation and concentrated in a 10 kD ultra-filtration column (Millipore, Burlington, MA, USA). The concentrated scFv antibody solution was diluted ten times with binding buffer (20 mM PBS, 40 mM imidazole, pH 7.4). The protein was purified with a HisTrap HP column and eluted with elution buffer (20 mM PBS, 500 mM imidazole, pH 7.4). Protein concentration was determined using the BCA protein assay kit.

### 2.7. Western Blot

The purified scFv antibodies were determined by western blot. After SDS-PAGE, the gel was transferred to the PVDF membrane (350 mA, 30 min) and then blocked with 5% skim milk at room temperature for 1 h. The anti-his and anti-Myc mouse antibodies (CWBIO, Taizhou, China) with a dilution of 1:1000 were added, respectively, as the primary antibodies and incubated at room temperature for 1 h. The membrane was washed 3 times with TBST (50 mM Tris-HCl, 100 mM NaCl, 0.5% Tween-20). The goat anti-mouse IgG secondary antibody (CWBIO, China) with 1: 5000 dilution was added and incubated at room temperature for 1 h. After washing 3 times with TBST, the membrane was detected using an ECL substrate (Tanon, Shanghai, China).

The binding affinity of the antibody with purified HpaA was also determined by western blot. The membrane was incubated with purified scFv antibody (2 μg/mL) as the primary antibody and 1:4000 diluted HRP-conjugated mouse anti-Myc antibody (Genscript, Zhenjiang, China) as the secondary antibody.

For the specificity and cross-reactivity test, nine *H. pylori* strains and seven different species existing in human feces, including *Achromobacter xylosoxidans*, *Enterococcus faecium*, *Enterococcus faecalis*, *Arcobacter cryaerophilus*, *Enterobacter cloacae*, *Vibrio mimicus*, and *Campylobacter jejuni*, were selected. The whole bacterial proteins of each strain were obtained by boiling, and the western blot experiment was carried out according to the above method.

### 2.8. Affinity of scFv A8 Antibody

The affinity of the scFv A8 antibody was determined by ELISA. Briefly, 96-well plates were coated with HpaA protein diluted to different concentrations (0.25 μg/mL,0.5 μg/mL, and 1 μg/mL, respectively) overnight at 4 °C. After washing 3 times with 0.05% PBST, the plates were blocked with 5% skim milk at 37 °C for 1 h, then incubated with serially diluted antibodies (from 15 μg/mL to 7 ng/mL) at 37 °C for 1 h. Plates were washed 5 times, followed by adding 1:1000 diluted HRP-conjugated anti-Myc mouse antibody and incubated at 37 °C for 1 h. The bound antibody was detected using the TMB substrate solution, and absorbance at 450 nm was determined with a microplate reader (BioTek, Santa Clara, CA, USA).

The binding kinetic of scFv A8 was determined using the Biolayer Interferometry (BLI) method with the Octet R8 System. The HpaA was incubated with 10 μM biotin on ice for 2 h, and excess biotin was removed with a PD MiniTrap™G-25 Desalting Column (Cytiva, Wilmington, DE, USA). Biotinylated HpaA (5 μg/mL) was loaded onto supper streptavidin biosensors (SSA, Sartorius, Gottingen, Germany), and diluted antibody (100 nM) was added for an association step of 200 s followed by a dissociation step of 300 s. The data were analyzed using ForteBio data analysis 12.0.

## 3. Results

### 3.1. Construction of scFv Antibody Library

**Figure 1:**
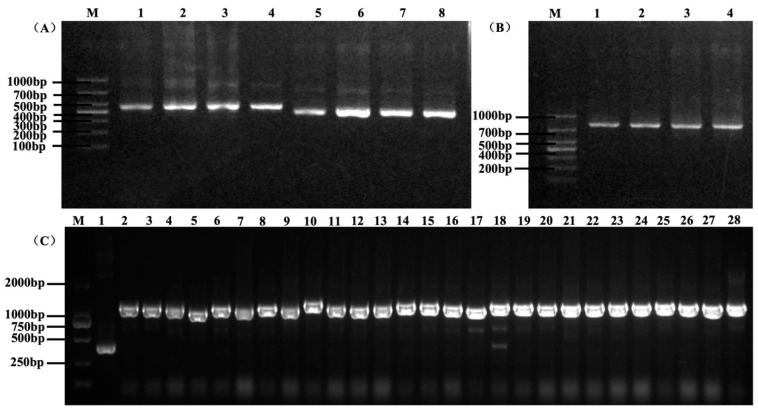
Amplification of V_H_, V_L_, and scFv fragments by PCR. (**A**) The PCR products of V_H_, V_L_. M, and DNA marker. Lanes 1–4 indicate V_H_ fragments with a size of 400 bp. Lanes 5–8 indicate V_L_ fragments with a size of 360 bp. (**B**) The PCR products of scFv. Lanes 1–4 indicate scFv fragments with a size of 800 bp. (**C**) Partial PCR results of the selected 47 clones. The correct size of the insert sequence was approximately 1200 bp. M indicates a DNA marker. Lane 1 indicates the pHEN2 plasmid sequence (negative control) with a size of 400 bp. Lanes 2–28 indicate fragments of different clones selected.

**Figure 2:**
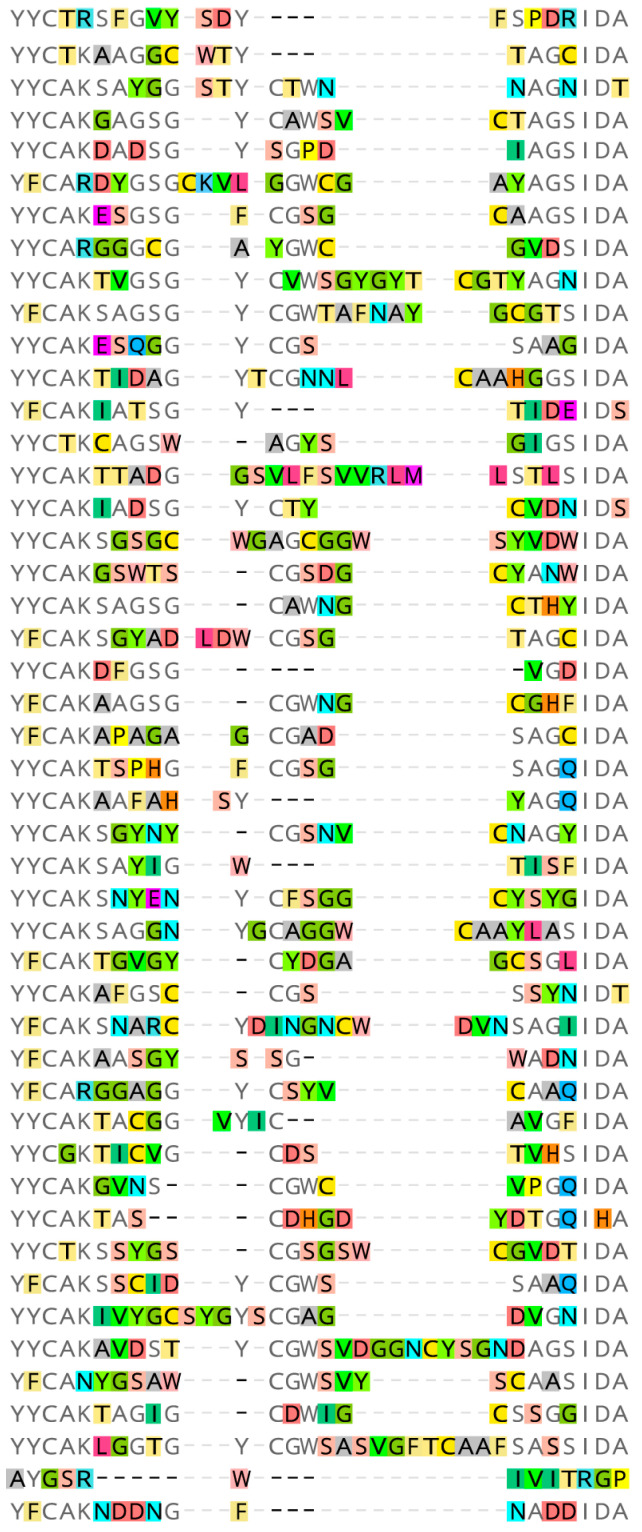
Sequencing results of scFv from 47 different clones. The sequences aligned were CDRH3 regions, which were responsible for recognizing specific antibody epitopes, indicating the diversity of the antibody library.

The PCR products with the correct sizes of 400 bp and 360 bp, corresponding to V_H_ and V_L_ domains, were amplified from the total RNA of five immunized chickens (Figure 1A). A 15-aa linker connected the VH and VL domains to make the full-length scFv, which was approximately 800 bp (Figure 1B). The scFv fragments were cloned into the pHEN2 vector, and the primary scFv antibody library size was estimated to be 1 × 10^9^ cfu/mL based on the dilution counting method. PCR analysis of 47 clones randomly selected from the antibody library showed that approximately 100% of the clones carried an insert of the correct size (Figure 1C), and the sequences of these PCR products were different (Figure 2), indicating the good diversity of our antibody library.

After infection by the M13KO7 helper phage, the titer of the phage display library was measured as approximately 1 × 10^13^ pfu/mL, and the displaying scFv antibodies were used for the subsequent selection of specific antibodies.

### 3.2. Expression and Purification of Recombinant HpaA Protein

**Figure 3:**
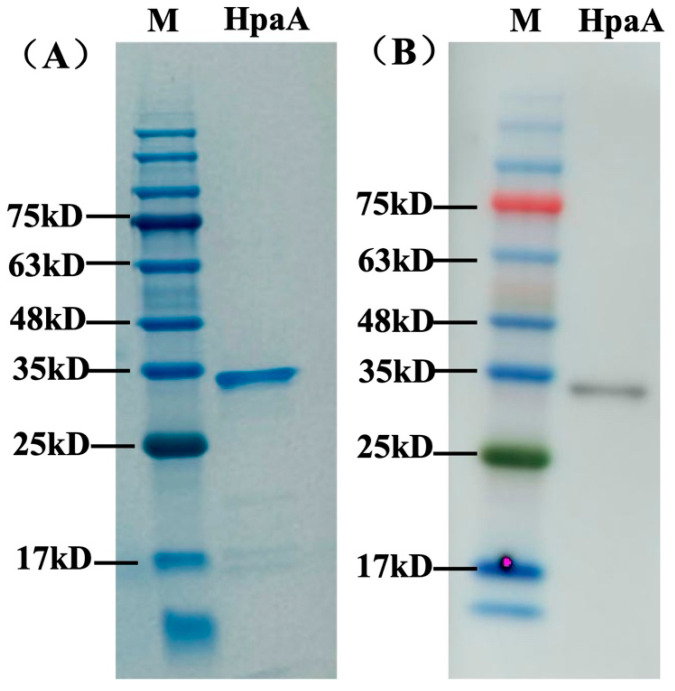
The expression and western blot analysis of HpaA. (**A**) The purification of HpaA by a HisTrap HP column. A band at 33 kD was obtained for HpaA. (**B**) The binding of antibody A8 against HpaA. The antibody A8 recognized HpaA at a molecular mass of 33 kD. M indicates a protein marker.

The extracellular domain (residues 28–260) of the HpaA protein was expressed in our study. The fragment of the *hpaA* gene was cloned into the pET-28a vector and expressed the His-tagged recombinant protein. SDS-PAGE results showed that the HpaA protein was successfully expressed with an expected molecular mass of 33 kD. After purification by a HisTrap HP column, a highly purified (>90%) and soluble protein was obtained (Figure 3A), which could be used as an antigen for screening antibodies.

### 3.3. Selection of Anti-HpaA scFv Antibodies

We screened the scFv phage library to obtain antibodies that specifically bound HpaA by bio-panning. Three rounds of panning against HpaA were performed to enrich specific binders. The ratios of output and input phages increased after each round of panning (Table 2), indicating enrichment of specific antibodies. After 3 rounds of panning, the phage libraries had been enriched 167 times (1 × 10^9^/6 × 10^6^).

After three rounds of panning, 196 clones from the panned library were randomly selected to obtain high-affinity scFv antibodies and analyzed by phage ELISA. More than 50% of the clones had a positive reaction against HpaA, and 26 clones with significant signals were further selected for sequence analysis. The framework regions of these antibodies were conserved and consistent with the chicken antibody sequences in the NCBI database. As shown in Figure S1, the sequences of complementarity determinative regions (CDRs) were variable, especially in CDRH3. Overall, 21 sequences with 10 different CDRH3, which may recognize different antigen epitopes, were identified. We selected one scFv (named A8), which was one of the most enriched sequences for further assays.

### 3.4. scFv Antibody A8 Expression and Purification

In our study, the antibody A8 was first expressed using the pET28a expression vector and *E. coli* BL21(DE)_3_. However, the protein was insoluble. *P. pastoris* is widely used to express antibodies with high yield and good solubility characteristics. Therefore, we chose *P. pastoris* for the expression of the antibody. The scFv fragment was amplified using a plasmid containing antibody A8 sequence as a template and ligated into the pICZαA expression vector with an α-factor secretion signal sequence for secretory expression. After 3 days of culture, eight positive clones were selected for expression, and SDS-PAGE analyzed the supernatant of culture. As shown in Figure S2A, a band at 38 kD was observed after induction for 48 h. The clone with the highest expression level was selected for further expression in large volumes. After ultrafiltration of the supernatant and purification with a nickel column, about 95% protein purity was obtained (Figure S2B). The western blot results using anti-his and anti-Myc mouse primary antibodies confirmed the expressed proteins containing his and Myc tags (Figure S2C,D).

### 3.5. Binding Activity of scFv Antibody A8

The specific activity of purified scFv antibody A8 was analyzed against recombinant protein by western blot. As shown in Figure 3B, antibody A8 efficiently recognized recombinant protein HpaA with a band at 33 kD.

**Figure 4:**
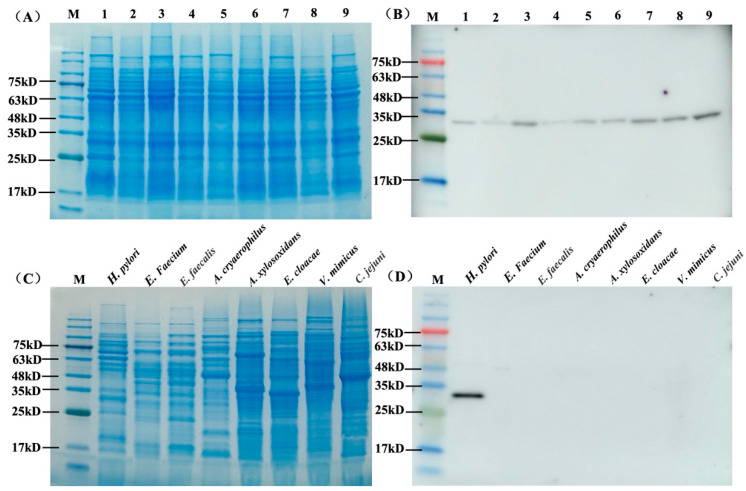
The binding of antibodies against various strains. (**A**) The SDS-PAGE of total proteins from *H. pylori* strains. M, protein marker. Lanes 1–9 indicate different *H. pylori* strains. (**B**) The western blot results of total protein from *H. pylori* strains. M, protein marker. Lanes 1–9 indicate different *H. pylori* strains. The band at 29 kD indicates HpaA protein. (**C**) The SDS-PAGE of total proteins from *H. pylori* and seven intestinal flora strains. (**D**) The western blot results of total protein from *H. pylori* and seven intestinal flora strains. M indicates a protein marker.

To further analyze the range specificity of the A8 antibody, lysates of nine various *H. pylori* clinical strains (Figure 4A) were selected to assess the specificity by western blot. As shown in Figure 4B, the antibody bound all strains tested with a band at 29 kD (the size of HpaA in *H. pylori*). The results suggested that the antibody had broad range specificity to various *H. pylori* strains and was highly specific for the respective antigen (no other proteins of *H. pylori* detected).

### 3.6. Cross-Activities of scFv Antibody A8

Cross-activities of scFv antibody A8 to other species isolated from feces were also tested with western blot. The lysates of seven various species were obtained by boiling and proceeded to SDS-PAGE (Figure 4C). Western blot analysis revealed that the antibody did not show binding with other species (Figure 4D).

### 3.7. scFv Affinity Determination

**Figure 5:**
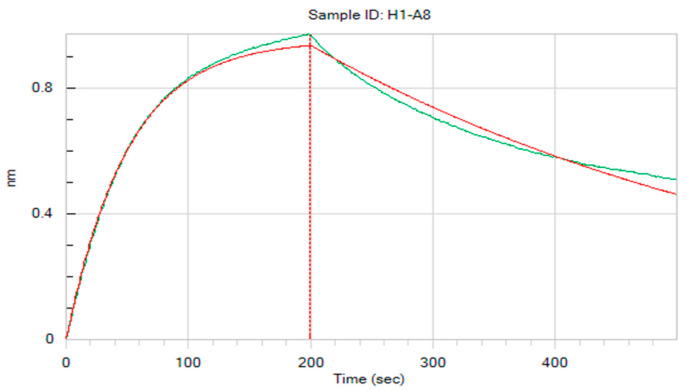
The binding of A8 to HpaA. The BLI binding kinetic of A8 antibody.

The binding affinity of scFv A8 to HpaA was evaluated using ELISA and BLI methods. The result of the ELISA assay demonstrated that antibody A8 bound well to recombinant HpaA with an EC_50_ of 32.3 nM. In addition, the kinetic rate constant of A8 was determined using BLI analysis. The result showed that antibody A8 exhibited a high affinity against HpaA (Figure 5), with an equilibrium dissociation constant (K_D_) of 13.3 nM, consistent with the ELISA assay.

## 4. Discussion

Most antibodies (mainly IgG) used to recognize *H. pylori* antigens were derived from mouse or rabbit. Antibodies from mouse could interact with many mammalian and bacterial proteins, limiting their use in diagnostic approaches. Due to the phylogenetic distance, the production of antibodies was generally more successful in chicken than in other mammals. Immunization of chickens represents an excellent alternative to obtain antibodies specific to certain epitopes that may not be dominant in mammalian immune responses.

The chicken-derived antibodies are technically easier to generate and display specificity and affinity. IgY antibodies with high affinity against bacterial or human proteins have been developed. Our previous study revealed that Anti-*H. pylori* IgY derived from specific strain immunized chickens could recognize different antigens well [21]. In general, detection based on polyclonal antibodies obtained from animals immunized with whole-bacteria antigens of *H. pylori* increased the possibility of cross-reactivity with other bacteria. However, the specificity of IgY was comparable with monoclonal antibodies from mouse [12]. scFv possesses the characteristics of IgY as well as its remarkable characteristics, such as easy genetic manipulation to improve further affinity, large-scale preparation with *E. coli* or yeast expression systems, and batch-to-batch consistency [22,23,24]. In addition, the development of phage display, a cost-effective method compared to hybridoma that avoided the need for repeatedly immunizing chickens [25], made the scFv easy to obtain and enhanced the utility of chicken antibodies. Moreover, compared to the mouse immunoglobulin system, the simplicity of the chicken system contributes to the construction of antibody libraries relatively easily.

Few studies on scFv antibodies from murine or humans have been reported in *H. pylori*. A previous study reported a murine anti-*H. pylori* scFv recognized a 30 kD surface protein and could inhibit the growth of *H. pylori* in vitro [26]. Human scFvs against *H. pylori* urease and conserved domains of VacA were also generated [8,27]. In this study, we first constructed an immune chicken scFv library, which increased the possibility of obtaining specific and high-affinity antibodies against antigens.

For scFv, V_H_ and V_L_ regions can be freely recombined, allowing for the indefinite repetition of gene recombination, leading to the large size and diversity of the antibody library [28]. The parameters for antibody library quality included gene diversity, library size, and screening ability. The size of immune libraries constructed in other studies was usually 10^7^ cfu [12,24], and in our research, we successfully built an immune scFv antibody library with a size of 10^9^ cfu. In addition, the region containing the most differences was CDRH3, which was responsible for recognizing specific antibody epitopes, indicating the good diversity of our antibody library. Moreover, we used recombinant HpaA, an outer membrane of *H. pylori* [29], as a target antigen to screen scFv antibodies. The sequences of HpaA are highly conserved among *H. pylori* isolates [30] and exhibited limited sequence homology with other proteins [31]. After bio-panning, which was used to enrich the clones that bound to the target antigen, the antibodies specific for HpaA were successfully selected and demonstrated specific binding and high affinity to the recombinant HpaA protein as well as nature HpaA antigens in different *H. pylori* clinical strains. In addition, the screened antibody had no cross-reaction with other common intestinal flora, indicating the success of our library construction.

Currently, the stool antigen test (SAT), a non-invasive test for *H. pylori* detection [32,33], is suitable for adolescents and children and self-testing at home. Several commercial SAT detection kits used polyclonal or monoclonal antibodies obtained from rabbit and mouse [34,35]. Our antibody library provided an easier and more economical way to prepare specific antibodies for accurate diagnosis of SAT, which could develop and may be used in a promising method for *H. pylori* diagnosis. Further study is necessary to pair the high-affinity antibodies against HpaA and evaluate the potential use for *H. pylori* detection.

## 5. Conclusions

In this study, we reported a first analysis on constructing and validating a chicken-derived immune scFv antibody library against *H. pylori*. The established antibody library had a large capacity, good diversity, and good screening ability, which was validated through selection-specific and high-affinity scFv antibodies against recombinant HpaA and clinical *H. pylori* strains. Generating highly specific scFv antibodies using the phage display technique in chickens is feasible and efficient. This work provides a new approach to building a pool of antibodies for diagnosing and treating *H. pylori*.

## Figures and Tables

**Table 1:** The primers used in this study.

Gene	Primer
*hpaA*	F-CGCGGATCCATGTGCAGCCCGCATATTATT R-CGCCTCGAGTTATCGGTTTCTTTTGCCTTTTA
V_H_	F-GTCCTCGCAACTGCCCATGGCTGATGGCGGCGTGA R-CCGCCAGAGCCACCTCCACCTGAACCGCCTCCACCGGAGGAGACGATGACTTCGG
V_L_	F-TCAGGTGGAGGTGGCTCTGGCGGAGGCGGATCGGCGCTGACTCAGCCGTCCTCGG R-TGATGGTGGCGGCCGCATTGGGCTG
scFv-1	F-GCCGGCCATGGCTGATGGCGGCCGTGACG R-ATGTGCGGCCGCATTGGGCTGGCCTAGGACGGT
scFv-2	F-GCCGGGAATTCCTGATGGCGGCCGTGACG R-ATGTGCGGCCGCATTGGGCTGGCCTAGGACGGT
scFv-3	F-TGGAATTGTGAGCGGATAACAATT R-GTAAATGAATTTTCTGTATGAGG

F, forward primer. R, reverse primer. The sequences underlined indicate the restriction enzyme sites.

**Table 2:** Phage input and output in each round of panning.

Round	Input (pfu)	Output (pfu)	Ratio (%)
1	9 × 10^12^	6 × 10^6^	6 × 10^−7^
2	1 × 10^12^	2 × 10^6^	2 × 10^−6^
3	1 × 10^12^	1 × 10^9^	1 × 10^−3^

pfu, plaque-forming unit.

## Data Availability

The raw data supporting the conclusions of this article will be made available by the authors on request.

## Supplementary Materials

The following supporting information can be downloaded at https://www.mdpi.com/article/10.3390/microorganisms12061148/s1, Figure S1. The sequencing results of anti-HpaA scFv antibodies from selected clones. Figure S2. The expression and identification of antibodies in *P. pastoris*.
