# Supplementary material for: Construction and Validation of Chicken Immune scFv Antibody Library against Helicobacter pylori
Source: Microorganisms. 2024 Jun 5;12(6):1148. doi: 10.3390/microorganisms12061148 (PMC11206095; doi:10.3390/microorganisms12061148)
Supplement: Supplementary file 1 [file microorganisms-12-01148-s001.zip › microorganisms-2958324-supplementary.pdf]

Figure S1. The sequencing results of anti-HpaA scFv antibodies from selected clones.

The sequences aligned were CDRH3 regions.

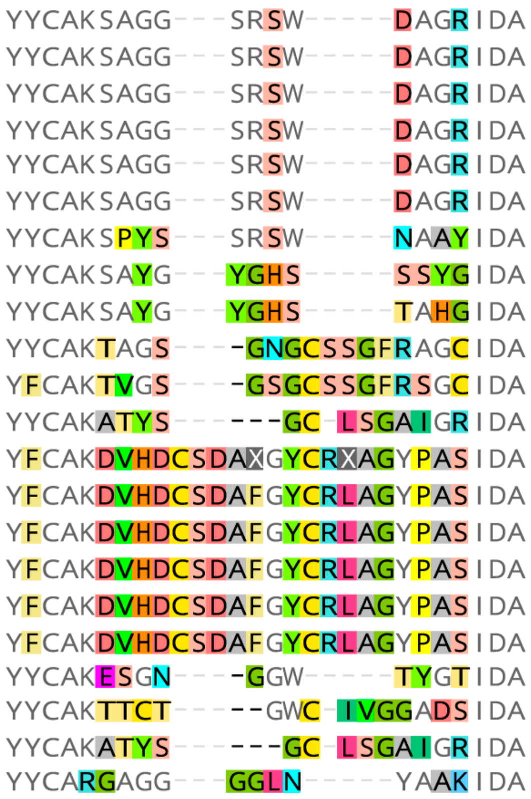

Figure S2. The expression and identification of antibodies in *P. pastoris*.

(A) The expression of scFv antibodies. M, protein marker. Lane 1-8 indicated different clones selected for expression. (B) The purification of scFv antibodies. M, protein marker. Lane 1-4 indicated different scFv antibodies. (C) The identification of scFv antibodies with anti-his antibody by western blot. 1, GS115 strain as negative control. Lane 2-9 indicated different positive clones. (D) The identification of scFv antibodies with anti-Myc antibody by western blot. Lane 1-8 indicated different positive clones.

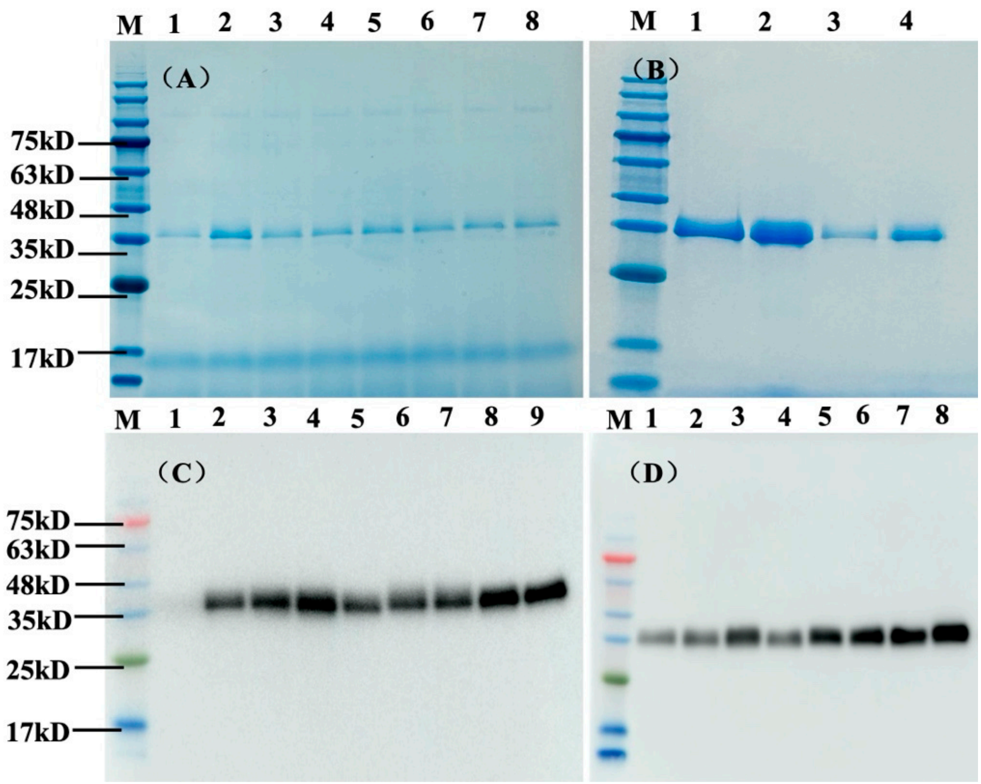

M 1 2 3 4 5 6 7 8
(A)
75kD
63kD
48kD
35kD
25kD
17kD
M 1 2 3 4
(B)
M 1 2 3 4 5 6 7 8 9
(C)
75kD
63kD
48kD
35kD
25kD
17kD
M 1 2 3 4 5 6 7 8
(D)
